# Malignant pancreatic serous cystic neoplasms: systematic review with a new case

**DOI:** 10.1186/s12876-016-0518-0

**Published:** 2016-08-22

**Authors:** Jimi Huh, Jae Ho Byun, Seung-Mo Hong, Kyung Won Kim, Jin Hee Kim, Seung Soo Lee, Hyoung Jung Kim, Moon-Gyu Lee

**Affiliations:** 1 Department of Radiology and Research Institute of Radiology, University of Ulsan College of Medicine, Asan Medical Center, 88 Olympic-Ro 43-Gil, Songpa-Gu, Seoul 05505 Korea; 2 Department of Radiology, University of Ulsan College of Medicine, Ulsan University Hospital, 877 Bangeojinsunhwando-ro, Dong-gu, Ulsan 44033 Korea; 3 Department of Pathology, University of Ulsan College of Medicine, Asan Medical Center, 88 Olympic-Ro 43-Gil, Songpa-Gu, Seoul 05505 Korea

**Keywords:** Serous cystadenocarcinoma, Serous cystic neoplasms, Pancreas, Computed tomography, Magnetic resonance imaging

## Abstract

**Background:**

This study analyzes the clinicopathologic and radiologic characteristics of malignant serous cystic neoplasm (SCN) of the pancreas through systematic review and an institutional case report.

**Methods:**

A comprehensive literature search was performed in the MEDLINE database to identify studies on malignant SCNs of the pancreas that had detailed clinicopathologic and radiologic information. A computerized systematic search of our institutional database was also performed to identify cases of malignant SCN for addition to the systematic review. Using the final included cases, we analyzed the clinicopathologic and radiologic features of malignant SCNs of the pancreas.

**Results:**

A review of 136 candidate articles identified 26 studies with 26 cases that had detailed clinical information. Our institutional data search added one case. The systematic review of the 27 cases revealed that primary tumors (mean diameter 10.2 ± 4.0 cm) mainly involved the body and tail of the pancreas (*n* = 16) and frequently invaded adjacent organs (*n* = 19). Distant metastases occurred in 14 patients (synchronous, *n* = 5; metachronous, *n* = 8; both, *n* = 1), most commonly in the liver (*n* = 13). Imaging features of malignant SCNs of the pancreas were identical to the benign counterpart, except local invasion or distant metastases. The prognosis was excellent in that 17 were alive at the time of writing with a median follow-up period of 2 years.

**Conclusions:**

The malignant potential of SCNs of the pancreas should be considered in the diagnosis and management of patients with pancreatic SCNs.

## Background

Serous cystic neoplasms (SCNs) of the pancreas are usually cystic epithelial neoplasms composed of cuboidal, glycogen-rich, epithelial cells that produce serous fluid [1].

Pancreatic SCNs represent approximately 10–16 % of all kinds of cystic pancreatic lesions [2–5]. Since Compagno et al. classified cystic neoplasms of the pancreas into serous and mucinous types in 1978 [6], SCNs were considered as a benign disease entity without risk of malignant transformation. This concept changed after George et al. reported the first case of a malignant SCN of the pancreas in 1989 [7]. Thereafter, the number of case reports or series of malignant SCNs of the pancreas increased [4, 8–31]. Thus, SCNs of the pancreas are not considered totally benign, but have an extremely low malignant potential. Regarding the incidence of malignant SCNs, the majority of literature reported less than 1 %, including the largest series which showed three serous cystadenocarcinomas from 2622 patients [32], even though a few literature reported 1–5 % [5, 18, 33]. Due to its very rare chance to be malignant, the vast majority of SCNs are safely observed without resection [34].

As a result of their extreme rarity, the characteristics of malignant SCNs of the pancreas were not well explored until the World Health Organization (WHO) classification published in 2010 established the definition of serous cystadenocarcinoma and described its characteristics [1]. In the 2010 WHO classification, malignancy is defined by the presence of distant metastases regardless of benign-looking histologic features. However, the WHO classification is still under debate as many studies have classified SCNs that invade surrounding organs as malignant SCNs even though there is no distant metastasis [4, 9, 12, 13, 16, 21, 22, 24, 25, 27, 29, 33]. Therefore, in this article, we use the broad term ‘malignant SCNs’ to include either SCNs with distant metastasis or local invasion.

Despite the increasing number of cases of malignant SCNs, there has been no systematic review to summarize the variable presentations of malignant SCNs and to provide a perspective of this rare disease entity. Therefore, we performed a systematic review of the malignant SCNs of the pancreas with the addition of our single case.

## Methods

### Systematic literature search

A computerized search of the MEDLINE database was conducted to find relevant studies published prior to April 30, 2015. Studies were eligible for inclusion if they described the clinicopathologic features, imaging findings, treatment, and outcome of the cases with regard to malignant SCNs of the pancreas. Any type of publication including case reports or case series was eligible. The following search terms were used: (pancreas OR pancreatic) AND (“serous cystadenocarcinoma” OR “serous cystic neoplasm”). Our search did not set any restriction or filter. To expand the search, the bibliographies of articles that remained after the selection process were screened for other potentially suitable articles.

### Institutional data search and case presentation

Our institutional review board approved the search of electronic medical records for this study. We performed a systematic computerized search of our institutional database (ABLE, Asan Medical Center) from January 1996 to April 2015 using the diagnostic codes of ‘serous cystic neoplasm of the pancreas’, ‘serous cystadenoma of the pancreas’, and ‘serous cystadenocarcinoma of the pancreas’. Using these search terms, we identified 447 patients initially diagnosed with SCN of the pancreas. Among the 447 patients, only one case was confirmed as serous cystadenocarcinoma of the pancreas. We obtained an informed consent from the patient. We present the clinical course, pathologic findings, and imaging features of this case. We also included this case in the systematic review of malignant SCNs.

### Analysis of clinicopathologic and radiologic features

For the malignant SCNs from the literature search and our institution, we analyzed their clinicopathologic features, patterns of metastasis and local invasion, treatment, and outcome. When imaging features of the primary tumors and/or metastatic tumors were available, we also analyzed these imaging findings.

## Results

### Literature selection

**Fig. 1:**
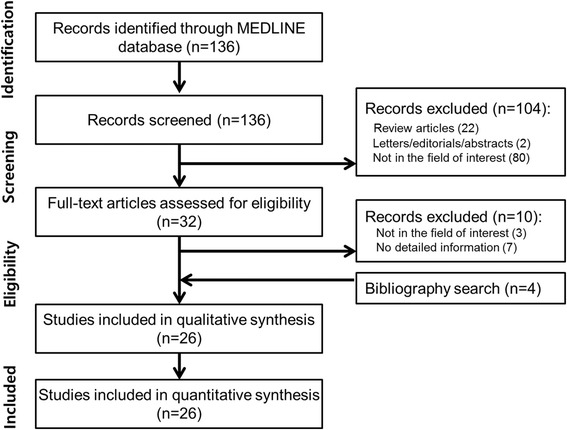
Flow diagram for literature selection

Our study selection process is shown in Fig. 1. The literature search in the MEDLINE database generated 136 initial candidate articles. After reviewing the titles and the abstracts and excluding 22 review articles, two conference abstracts, and 80 articles that were not in the field of interest for this study, 32 articles were initially selected for eligibility. The full text of the 32 articles was retrieved. The search of the bibliographies of these articles found four additional eligible studies. Among these 36 eligible studies with 42 cases, we further excluded three articles that were not in the field of interest and seven studies that did not have detailed information on pancreatic serous cystadenocarcinomas, and selected 26 studies with 26 cases [4, 7–31]. We also included the present case from our institution; thus finally 27 cases were reviewed in this systematic review.

### Presentation of our case

**Fig. 2:**
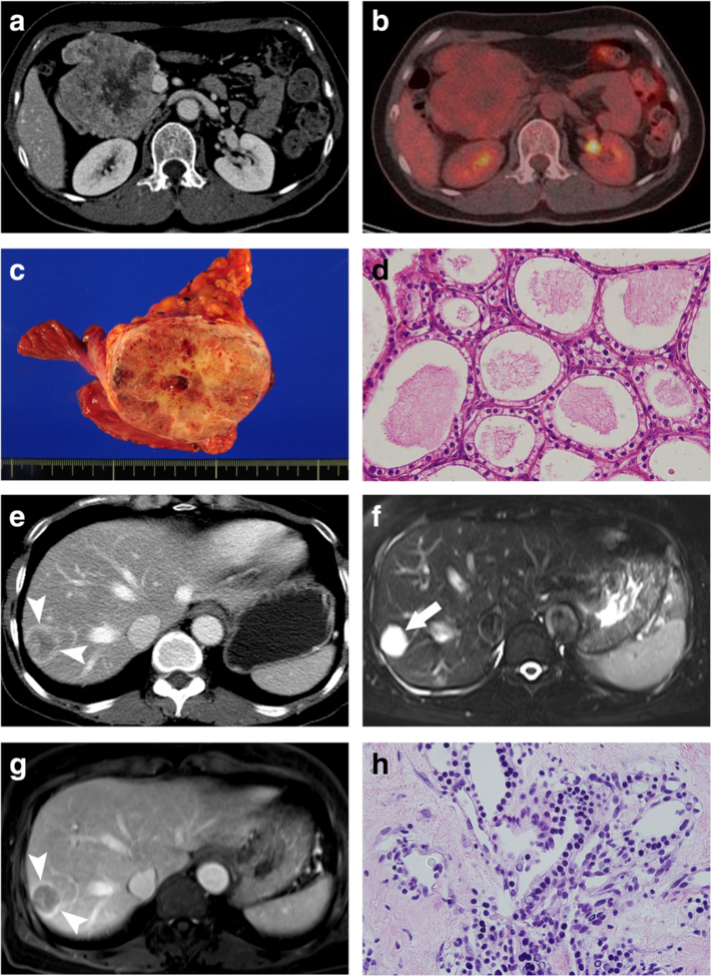
A 52-year-old woman with malignant serous cystic neoplasm of the pancreas and metachronous hepatic metastasis. **a** Contrast-enhanced CT scan demonstrates a 9 × 8 cm solid and cystic mass in the pancreatic head. **b** FDG-PET/CT scan demonstrates no increased uptake within the tumor. **c** Photograph of the gross specimen shows a well-demarcated lobulated mass. The cut surface showed a yellowish, gray, firm tumor with multiple microcystic changes and fibrous septa. **d** Photomicrograph shows multiple microcysts lined by a single layer of cuboidal epithelium with a clear cytoplasm (H and E stain, ×200). **e** Contrast-enhanced CT scan shows a low-attenuating nodular lesion with peripheral rim enhancement in liver segment VII. **f** On T2-weighted MR image, the liver tumor shows very bright signal intensity like cerebrospinal fluid. **g** On contrast-enhanced T1-weighted MR image during the portal-venous phase, the tumor shows peripheral rim enhancement. **h** Photomicrograph shows that the tumor is composed of multiple microcysts separated by collagen fibers, which are lined by a single layer of cuboidal epithelium with a clear cytoplasm and bland-looking nuclei (H and E stain, ×200)

A 52-year-old woman presented to our institution with a palpable abdominal mass. On physical examination, a large, soft mass was palpable in the epigastrium. Results of laboratory examinations and tumor markers (carcinoembryonic antigen and carbohydrate antigen 19–9) were within the normal range. Serum chromogranin A was 108 ng/mL, which was around the upper normal limit (27–94 ng/mL). Contrast-enhanced computed tomography (CT) scans showed a 9 × 8 cm solid and cystic mass in the head of the pancreas (Fig. 2a). The peripheral portion of the tumor was well enhanced, while the central portion was not well enhanced. On ^18^F-fludeoxyglucose (FDG) positron emission tomography (PET)/CT scans, the tumor did not show any increased uptake (Fig. 2b).

Pancreaticoduodenectomy was performed with a clinical diagnosis of a solid pseudopapillary neoplasm or pancreatic neuroendocrine tumor. On the gross specimen, a well-demarcated lobulated mass was observed in the pancreas head. The cut surface showed a yellowish, gray, firm tumor with multiple microcystic changes and fibrous septa (Fig. 2c). Vascular and perivascular invasions and nodal involvement were not observed. Microscopic findings revealed multiple microcysts separated by collagen fibers. The inner surface of the cysts was lined by a single layer of cuboidal epithelium with a clear cytoplasm (Fig. 2d). No mitoses or cellular atypia were noted. Immunohistochemical staining was positive for the cytokeratins, AE1 and AE3, and negative for CD56, chromogranin A, synaptophysin, renal cell carcinoma marker, and CD10. The epithelial cells of the tumor had cytoplasmic periodic acid-Schiff (PAS)-positive granules. The histopathological diagnosis was serous cystadenoma of the pancreas.

Five years later, the patient presented with abdominal discomfort. On contrast-enhanced CT, there were multiple liver masses/nodules that showed peripheral rim enhancement (Fig. 2e). Under suspicion of liver metastases, FDG-PET scanning was performed, which revealed no FDG avidity in any liver masses/nodules. There was no evidence of local tumor recurrence or extrahepatic metastasis on FDG-PET/CT scans and body CT. The tumor showed very high signal intensity (SI) like cerebrospinal fluid on T2-weighted magnetic resonance (MR) image (Fig. 2f) and low SI on T1-weighted image. On diffusion-weighted images and apparent diffusion coefficient map, there was no diffusion restriction in the tumor. On multiphasic contrast-enhanced T1-weighted MR images, the tumor showed peripheral rim enhancement in the arterial phase, portal-venous phase, and delayed phase (Fig. 2g).

Ultrasound-guided biopsy was performed for the liver masses. Pathologic results showed that the tumor specimen was composed of multiple microcysts separated by collagen fibers (Fig. 2h). The microcysts were lined by a single layer of cuboidal epithelium with a clear cytoplasm and bland-looking nuclei. These histopathologic features were very similar to those of the previous pancreaticoduodenectomy specimen. Finally, the histologic diagnosis was confirmed as serous cystadenocarcinoma with metachronous liver metastasis.

The patient has been observed without any treatment for the liver metastases for 17 months after liver mass biopsy. The liver metastases have minimally enlarged on follow-up CT as the largest liver metastasis has increased from 3.1 to 3.4 cm in the longest diameter over 17 months. However, the patient has been asymptomatic and is still alive at the time of writing.

### Systematic review

The characteristics of the 27 included cases of malignant SCNs are summarized in Table 1. The 27 cases were diagnosed as malignant SCNs of the pancreas because they exhibited characteristics of malignancy including local invasion and/or distant metastasis. According to the definition of the 2010 WHO classification, which defines pancreatic serous cystadenocarcinoma by the presence of distant metastases, only 14 cases were pancreatic serous cystadenocarcinoma. However, we also analyzed the cases with local invasion.

**Table 1:** Characteristics of included cases of malignant serous cystic neoplasms of the pancreas

Author	Year	Age	Sex	Signs or symptoms	Location	Tumor diameter (cm)	Gross morphologic patten	Distant metastatic sites	Synchronous/Metachronous^a^	Local invasion sites	Surgical therapy	Outcome
George et al	1989	70	M	Hemorrhage from gastric varices	body and tail	11	Micorocystic	Liver	Synchronous	Stomach, spleen, splenic vein	DP	Death during operation due to hemorrhage
Friedman et al.	1990	74	F	Right flank pain, weight loss, palpable mass	head	19	Macrocystic	Liver, lung, bone, adrenal glands	Synchronous	None	None	Death due to advanced neoplasm
Kamei et al.	1991	72	F	Jaundice	Multifocal origin (head, body,tail)	10	Microcystic			Neural invasion	TP	NA
Okada et al.	1991	63	F	Abdominal pain	body and tail	12	Microcystic	Liver	Metachronous (4 years)	None	DP	Alive 5 years after initial operation
Yoshimi et al.	1992	63	F	Epigastric pain, palpable mass	body and tail	12	Microcystic	Liver	Metachronous (3 years)	None	DP	Alive 6 years after initial operation
Ohta et al.	1993	64	M	Unrelated incidental detection of the tumor on abdominal CT	body	2.5	Microcystic			Perivascular and vascular invasion	Enucleation	Alive 9 months after initial operation
Widmaier et al	1996	71	M	Elevated liver function tests	head	4	Mixed			Peripancreatic fat	PPPD	Alive 1 year later
Ishikawa et al.	1998	63	F	Abdominal pain	body	12	Microcystic	Liver	Metachronous (3 years)	None	DP	NA
Eriguchi et al.	1998	65	F	Palpable abdominal mass	body and tail	16	Microcystic	Liver	Both (9 years)	None	DP, micorowave coagulo-necrotic therapy	Alive 10 year after initial operation
Abe et al.	1998	71	F	Palpable abdominal mass, general fatigue, weight loss	body and tail	12	Microcystic			Lymph node, peripancreatic fat	DP, splenectomy	Alive 2 years later
Horvath et al.	1999	81	F	NA	NA	6	NA			Vessel	DP	NA
Wu et al.	1999	57	F	Unrelated, incidental detection of the tumor on abdominal CT	entire pancreas	5.5	Mixed	Liver, peritoneum	Metachronous (10 year)	Stomach	Pancreatectomy, splenectomy, cholecystectomy	Recurrence 10 years after initial tumor resection
Strobel et al.	2001	56	F	Recurrent abdominal pain, diarrhea, weight loss	entire pancreas	14	Microcystic	Liver	Metachronous (3 years)	None	PPPD	Alive 3 years after initial operation
Matsumoto et al.	2004	87	F	no symptom, incidental finding(inguinal hernia op)	body and tail	12	Microcystic	Spleen	Synchronous	Spleen, Vessel	DP with colectomy	Uneventful (10 months)
Schintaku et al.	2005	85	F	Fatigue, intermittent diarrhea	body and tail	12	Microcystic			Spleen	DP, distal gastrectomy	Alive 10 months later
Friebe et al.	2005	80	F	Abdominal pain, anorexia, weight loss	body and tail	8	Microcystic			Spleen	DP, splenectomy	Alive 1 year later
Gupta et al.	2008	42	F	Abdominal pain, palpable abdominal mass, diarrhea, weight loss	body and tail	10	Macrocystic			peripancreatic fat.	DP, splenectomy	Alive 2 years later
Franko et al.	2008	68	F	Flank pain, weight loss, anemia, duodenal bleeding	head	5	Microcystic	Liver	Metachronous (3 years)	Splenoportal vein, duodenum	Inoperable	Death due to advanced neoplasm, 45 months later
King et al.	2009	70	M	Abdominal pain, hematemesis	head	9	Microcystic			Duodenum	PPPD	Alive 7 years later
Vadala et al.	2010	74	M	NA	head	NA	NA			Portal vein	PD, portal vein thrombectomy	NA
Bano et al.	2011	62	M	Abdominal pain, vomiting, weight loss, jaundice	head	7	Microcystic	Liver	Metachronous (1 year)	Duodenum	PD, microwave coagulo-necrotic therapy	Alive 1 year later
Cho et al.	2011	64	F	Dizziness, hematochezia	tail	12	Microcystic			Colon, spleen invasion	DP, segmental resection of the colon, splenectomy	NA
Bramis et al.	2012	86	F	Abdominal pain	body	17	Microcystic	Liver	Synchronous	Stomach	Inoperable, Biopsies taken	Died 1 month later due to unrelated other medical problem
Wasel BA et al.	2013	68	F	Incidental finding	tail	12	Microcystic			Liver, retroperitoneum	Inoperable, neo-adjuvant chemotherapy	Alive 1 year later
Rathore MU et al.	2013	60	F	Upper abdominal pain	body	9	Microcystic			Vessels	Partial pancreatectomy and splenectomy	Death 3rd post operative day due to thromboembolism
Kainuma O et al.	2015	69	M	Upper abdominal discomfort	body	6	Solid variant	Liver	Synchronous	None	Distal pancreatectomy(initial diagnossi) + Liver metastatectomy(27 months later)	Alive 30 months later
Present case	2015	52	F	Palpable mass	head	9	Microcystic	Liver	Metachronous (5 years)	None	PD	Alive 6.5 years later

*DP* distal pancreatectomy, *TP* total pancreatectomy, *PD* pancreaticoduodenectomy, *PPPD* pylorus preserving pancreaticoduodenectomy

^a^Number in parenthesis is time interval between detection of primary pancreatic tumor and metachronous metastasis

#### Clinical features

The mean age of patients with malignant SCNs was 68.0 ± 10.4 years with a range of 42–87 years. The female to male ratio was 2.9 (20 females and seven males). Regarding the presenting symptoms or signs, abdominal/flank pain was the most commonly presented (*n* = 13; 48.1 %), followed by a palpable mass (*n* = 6; 22.2 %), weight loss (*n* = 5; 18.5 %), gastrointestinal bleeding (*n* = 3; 11.1 %), incidental detection (*n* = 3; 11.1 %), and jaundice or abnormal levels of serum liver enzymes (*n* = 3; 11.1 %).

#### Morphologic and radiologic features

On gross specimens, the most common pattern was the microcystic type (*n* = 20), which is defined as multiple cysts measuring <2 cm separated by thin fibrous septa. The macrocystic type, which is composed of large cysts ≥2 cm, was observed in two cases. The mixed microcystic and macrocystic type was found in two cases. There was only one case with the solid type. In two articles, there was no information on the gross morphological type of SCN.

CT or MR imaging features were available in 20 cases, in which the imaging features of the primary tumors and/or metastatic lesions were qualitatively described. In all cases, the imaging findings of the malignant SCNs of the pancreas were basically identical to those of benign serous cystadenoma, except for malignant behavior such as local invasion or distant metastases. The radiologic features of serous cystadenoma or cystadenocarcinoma generally varied according to the gross morphologic pattern. Tumors with the microcystic pattern were generally shown as well-circumscribed, multilocular masses, with or without central stellate scars. The microcysts were not clearly identified on CT, but were very hyperintense on T2-weighted MR images. Tumors with the macrocystic pattern were shown as well-circumscribed, lobulated masses containing a few cysts. The SCNs with a mixed microcystic and macrocystic type simultaneously showed findings of the micro- and macrocystic types. The solid type of malignant SCN reported in one case was shown as a solid enhancing mass that initially mimicked a neuroendocrine tumor.

Regarding the malignant features, local invasion to the adjacent vessels, spleen, liver, adrenal glands, stomach, or colon could be detected on CT and/or MR images. Among the 27 cases with malignant SCNs, 13 had hepatic metastasis. Seven of thirteen cases described enhancement patterns and morphological characteristics of the liver metastasis. Six synchronous or metachronous metastases in the liver were cystic masses with peripheral enhancement on contrast-enhanced CT/MRI and showed very high SI on T2-weighted images. Only one hepatic metastatic mass showed heterogeneous wall enhancement; however, it also demonstrated very high SI on T2-weighted images like the other masses. Taking all into account, the liver metastases, which are basically cystic masses, can be characterized as exhibiting very high SI on T2-weighted images, with an enhancing cyst wall and/or fibrous septa.

#### Primary tumors and local invasion

The mean diameter of the primary pancreatic tumors was 10.2 ± 4.0 cm (range, 2.5–19 cm). The locations of the primary tumors were as follows: pancreatic head (*n* = 7; 25.9 %), pancreatic body and tail (*n* = 9; 33.3 %), pancreatic body (*n* = 5; 18.5 %), pancreatic tail (*n* = 2; 7.4 %), entire pancreas (*n* = 3; 11.1 %), or location unavailable (*n* = 1; 3.7 %).

Local invasion of the adjacent organs occurred in 19 patients (70.4 %) including six patients with invasion of multiple organs, as follows: adjacent vessels (*n* = 7; 25.9 %), spleen (*n* = 5; 18.5 %), peripancreatic fat (*n* = 3; 11.1 %), stomach (*n* = 3; 11.1 %), duodenum (*n* = 3; 11.1 %), colon (*n* = 1; 3.7 %), liver (*n* = 1; 3.7 %), retroperitoneum (*n* = 1; 3.7 %), and perineural invasion (*n* = 1; 3.7 %).

#### Metastasis patterns

Among the 27 cases with malignant SCNs, 14 had a distant metastasis: synchronous metastases in five patients (18.5 %), metachronous metastases in eight patients (32 %), and both synchronous and metachronous metastases in one patient (3.7 %). The time interval between detection of pancreatic SCN and metachronous metastases was from 1 to 10 years (median, 3 years).

Among the 14 patients with distant metastases, including two patients with metastasis in multiple organs, the sites of distant metastases were as follows: liver (*n* = 13; 92.9 %), lung (*n* = 1; 7.1 %), bone (*n* = 1; 7.1 %), adrenal glands (*n* = 1; 7.1 %), peritoneum (*n* = 1; 7.1 %), and spleen (*n* = 1; 7.1 %). Notably, liver metastasis was found in all patients with metachronous metastases. Among the 27 patients, only four patients had lymph node metastases; however, the exact site of lymph node metastasis was unavailable.

#### Treatment and outcome

Regarding the treatment of primary tumors, surgical resection of the primary pancreatic SCN was performed in 23 patients (85.2 %). Three patients were inoperable due to local invasion (*n* = 2) and synchronous metastases (*n* = 1). In one case report focusing on histopathologic features, the treatment was not stated [8].

Among the 27 cases, death was reported in five patients only, which was due to either an advanced pancreatic neoplasm (*n* = 2), perioperative mortality (*n* = 2), or unrelated medical problem (*n* = 1). The two patients who died from advanced tumors had metastasis in various organs. Time to death ranged from 3 days to 45 months from the initial surgery or diagnosis. In 17 cases, patients were alive at the time of writing, with a median follow-up period of 2 years (range, 9 months to 10 years from the initial surgery or diagnosis). Patient outcomes were not reported for the other five patients.

## Discussion

Our systematic review of malignant SCNs of the pancreas adds to growing evidence that SCNs of the pancreas have malignant potential. As a result of their rarity, radiologists and clinicians generally consider SCNs of the pancreas as benign. With the growing number of malignant SCNs, we need to establish diagnostic criteria for malignant SCNs of the pancreas. Regarding the primary tumors, malignant transformation into cystadenocarcinoma is suggested by an increase in size, heterogeneous morphological characteristics, and poor distinction from the surrounding pancreatic parenchyma [26]. Direct invasion of the adjacent organs and the presence of distant metastases are hallmarks of malignant SCNs of the pancreas. Although, the 2010 WHO classification defined malignancy by the presence of distant metastases regardless of benign-looking histologic features, the WHO classification is still under debate as many studies have classified SCNs that invade surrounding organs as malignant SCNs [4, 9, 12, 13, 16, 21, 22, 24, 25, 27, 29, 33]. However, it is difficult to differentiate local invasion from mass effect in a large tumor, particularly on preoperative imaging studies. Particularly, it is more difficult to identify the presence of local invasion in patients with SCNs as SCN is a cystic tumor. Therefore, the definition of local invasion in SCNs on imaging studies should be more clearly established like that of vascular invasion in solid tumors. The vascular invasion in solid tumors is usually defined as an encasement of the vessel (greater than 180° vascular circumferential involvement) or irregular narrowing of the vessel with involvement of vessel wall by the tumor [35].

Interestingly, in nine cases with metachronous distant metastasis, liver metastases occurred after complete resection of the primary tumor with the time interval ranging from 1 to 10 years. Therefore, follow-up imaging exams should include the liver. The liver metastases from serous cystadenocarcinoma are frequently cystic in nature with a similar histology to that of the primary pancreatic tumor. T2-weighted images might be the most important sequence for characterizing liver metastases. When new multiseptated cystic lesions are detected in the liver after resection of SCNs of the pancreas, the possibility of metachronous liver metastases should be considered.

Synchronous liver metastases occurred in five cases when the primary pancreatic SCNs were diagnosed. The imaging features of liver metastases were similar to those of primary pancreatic SCNs. Therefore, when multiseptated cystic lesions compatible with SCNs are found in the liver and pancreas, we should consider the possibility of pancreatic SCNs with synchronous liver metastases from the initial diagnostic step, despite its rarity. In these cases, a more vigorous diagnostic work-up is needed.

Regarding the outcome of those included studies, two died from perioperative mortality, one died from unrelated medical problem, and two died from advanced tumors. The other 17 patients were alive at the time of writing. These findings indicate that surgical resection should be decided very carefully due to high perioperative mortality and relatively indolent course of malignant SCNs. In current status, it is difficult to find predictive factors for death due to small number of death cases, warranting further evidence.

Nowadays, clinicians have a dilemma in the management of SCN of the pancreas [35]. In general, the current management of serous cystadenomas of the pancreas is essentially conservative. Indeed, the vast majority of cases do not need surveillance after initial diagnosis. Surgery is indicated in cases with new symptoms or complications such as abdominal pain, pancreatitis, and biliary obstruction [28]. Since the malignant potential in the pancreatic SCNs is very low, pancreatic SCNs can be safely observed without surgical resection in the vast majority of cases. However, due to its malignant potential, we need to establish certain criteria for the surveillance of SCN of the pancreas. A comprehensive panel of patient’s symptoms/signs, imaging, cytopathology, tumor growth rate, and biological activity is essential for decision making. Thus, regular physical exam, serum tumor markers, imaging studies including CT or MRI, endoscopic ultrasonography, and cyst fluid analysis should be performed appropriately.

Our study has limitations. First, it is a systematic literature review, hence the data such as malignant imaging features or local invasion were extracted from individual studies. The presence of malignant imaging features or local invasion were determined based on the imaging description in individual studies. Second, all cases were gathered from different institutions where the clinical practice might vary, which may raise issue of heterogeneity of included cases. However, this is also an inevitable limitation of a systematic literature review.

## Conclusions

In conclusion, the number of reported cases of malignant SCNs of the pancreas is growing, prompting radiologists and clinicians to redefine this disease entity and establish guidelines for diagnosis and management of malignant SCNs of the pancreas. A comprehensive panel of patient’s symptoms/signs, imaging, cytopathology, tumor growth rate, and biological activity is essential to diagnose and manage malignant SCNs of the pancreas.

## Abbreviations

CT: Computed tomography
DP: Distal pancreatectomy
FDG: ^18^F-fludeoxyglucose
MR: Magnetic resonance
PAS: Periodic acid-schiff
PD: Pancreaticoduodenectomy
PET: Positron emission tomography
PPPD: Pylorus preserving pancreaticoduodenectomy
SCN: Serous cystic neoplasm
SI: Signal intensity
TP: Total pancreatectomy
WHO: World Health Organization
